# How Well Do Videos on YouTube Meet Information Needs of ADRD Family Caregivers? A Qualitative Analysis

**DOI:** 10.1093/geroni/igaa057.875

**Published:** 2020-12-16

**Authors:** Shinyi Wu, Katherine Foster, Magaly Ramirez, Haomiao Jin, Elizabeth Joe

**Affiliations:** 1 University of Southern California, Los Angeles, California, United States; 2 University of Southern California, Fountain Valley, California, United States; 3 University of Washington, Seattle, Washington, United States; 4 Keck School of Medicine, USC, Los Angeles, California, United States

## Abstract

Family caregivers need access to information, education, and support for taking care of individuals with Alzheimer’s disease and other dementia (ADRD). As YouTube is increasingly being used for sharing health information, videos regarding the disease and caregiving are becoming an important source of information to support family ADRD caregivers. This study aimed to assess the types and quality of information available on YouTube videos related to ADRD caregiving. A framework categorizing information needs of family ADRD caregivers was developed through a qualitative study with caregivers (N=21) and with healthcare and community stakeholders (N=6). The framework was used to conduct content analysis of YouTube videos. Nineteen categories of information needs were identified, including information about ADRD, healthcare services and treatment, available community resources, caregiving skills, and short- and long-term care. YouTube videos that met the keywords, language, and view selection criteria were evaluated by two coders on a developed rating scale to measure their relevance and helpfulness. A neurologist verified the ratings in 10% of the coded videos for quality assurance. There were 48 English and 23 Spanish videos met the selection criteria. More English (89.6%) than Spanish (56.5%) videos provided tips on handling specific ADRD symptoms. The majority categories of information needs (15 of the 19) were absent in most videos (87.0%, 89.6%). Many of the most watched videos were not rated as helpful. Community-based providers and healthcare organizations are encouraged to make high quality needed information in commonly accessed videos sharing service to support ADRD family caregivers.

